# Why Do Physicians Prescribe Antibiotics? A Systematic Review of the Psycho-Socio-Organisational Factors Related to Potentially Inappropriate Prescribing of Antimicrobials in Europe

**DOI:** 10.3390/idr16040051

**Published:** 2024-07-25

**Authors:** Chiara Lansink, Bhanu Sinha, Nico Meessen, Tessa Dekkers, Nienke Beerlage-de Jong

**Affiliations:** 1 Section of Health Technology and Services Research, Technical Medical Centre, University of Twente, 7522 NB Enschede, The Netherlands; n.beerlage-dejong@utwente.nl; 2 University of Groningen, University Medical Center Groningen, Department of Medical Microbiology and Infection Prevention, Hanzeplein 1, 9713 GZ Groningen, The Netherlands; b.sinha@umcg.nl (B.S.); n.meessen@umcg.nl (N.M.); 3 Section of Psychology, Health and Technology, Centre for eHealth and Wellbeing Research, University of Twente, 7500 AE Enschede, The Netherlands; t.dekkers@utwente.nl

**Keywords:** potentially inappropriate prescribing, antibiotic resistance, antimicrobial prescribing, physicians, uncertainty

## Abstract

**Purpose:** Effective antimicrobial use enhances care quality and combats antibiotic resistance. Yet, non-guideline factors influence potentially inappropriate prescribing. This study explores psycho-socio-organisational factors in antimicrobial prescribing as perceived by physicians across primary, secondary, and tertiary care. **Methods:** Adhering to PRISMA guidelines, a systematic review was conducted using PubMed and Scopus databases from 1 January 2000, to 8 March 2023, with an update search until 30 January 2024. Inclusion criteria focused on studies in Europe exploring psycho-socio-organisational factors for antibiotic prescribing from physicians’ perspectives in hospital, inpatient, or primary care settings. Exclusion criteria targeted out-of-office prescriptions or low-quality studies. To evaluate the latter, several quality and risk-of-bias checklists were used. Data were extracted on study characteristics, study design, and methods and identified determinants of antibiotic prescribing. Data was analysed using a narrative synthesis method. **Results:** Among 8370 articles, 58 met inclusion criteria, yielding 35 articles from 23 countries. Three main themes emerged: personal, psychological, and organisational factors, encompassing seven determinants including work experience, knowledge, guideline adherence, uncertainty management, perceived pressure, time constraints, and diagnostic resource availability. Uncertainty management was key, with work experience and knowledge mitigating it. No additional factors emerged in the updated search. **Conclusion:** Enhanced uncertainty management decreases perceived patient and/or parental pressure to prescribe antibiotics, contributing to reducing potentially inappropriate prescribing (PIP). Therefore, it is imperative to educate physicians on effectively managing uncertainty. Interventions to improve antibiotic prescribing should be tailored to the specific needs and preferences of the different prescribing physicians.

## 1. Introduction

Effective antimicrobial therapy is a major and essential part of the medical treatment of many, often life-threatening conditions, that are primary infections or infectious complications of any medical or surgical intervention [1]. Appropriate and rational use of antimicrobials is crucial to improve quality of care [2,3,4], to limit collateral damage, both regarding side effects [5] and selective pressure for antimicrobial resistance (AMR) [4,6,7], and to achieve cost-effectiveness [8,9,10]. On a global scale, AMR is the most critical issue and is thus listed by the World Health Organisation as one of the top global public health threats [11]. There are numerous triggers for inappropriate and even irrational use of antimicrobials [12,13], with a substantial part of them being psycho-socio-organisational factors [14,15,16]. Antimicrobial stewardship aims at improving the quality of care and reducing (potentially) inappropriate prescribing (PIP) through a number of interventions [17,18], among others by improving knowledge on antimicrobial resistance among healthcare providers [19,20,21,22,23].

PIP refers to situations where antibiotics are prescribed unnecessarily or when the wrong kind, dose, or duration is prescribed [24]. Effectively improving appropriate prescriptions requires a behavioural change. However, it is well-known that interventions can only be successful if tailored to the problems, target group, and environmental context in which the behavioural change needs to occur [25]. Consequently, interventions to reduce PIP must account for and address all determinants of antimicrobial prescribing, including psycho-socio-organisational factors related to both patients and physicians [26,27]. Factors such as risk aversion, uncertainty avoidance, and patient demand may contribute to decision-making [28,29,30]. Additionally, prescribing behaviour is at least partly related to laws, regulations, and guidelines that apply within the specific country physicians are working in [31,32,33].

While psycho-socio-organisational factors may play a role, little is known about physicians’ perspectives on them. We hypothesised that physicians who are risk-averse dislike taking risks and, therefore, are probably more inclined to PIP. Uncertainty avoidance, defined as the degree to which an individual tries to cope with anxiety by minimising uncertainty, is expected to correlate with a higher likelihood [34,35]. In addition, patient behaviour may play a role. Patients explicitly requesting antibiotics receive prescriptions more often than those who do not make such requests [29,36,37]. This underscores the relevance of investigating how prescribing physicians perceive patient pressure and how this affects their behaviour. Finally, gaining insight into the prescribing physician’s perspective on the balance between “non-medical” and medical reasons for prescribing or refraining from antibiotics is crucial. Physicians, driven by a commitment to providing the best care for patients, may be unaware of instances of incorrect prescribing [29,38].

Since the Netherlands has a longstanding track record for antimicrobial stewardship, we have zoomed in on the inter- and intra-personal determinants of (potentially inappropriate) prescribing within this country in particular. Although the prevalence of AMR is comparatively lower than in many other European nations, there is room for improvement regarding antimicrobial prescriptions. Approximately 80–90% of all antibiotic prescriptions in the Netherlands originate from primary care [39]. Some general practitioners (GPs) prescribe antibiotics up to six times more frequently per 1000 patients than others, but the reasons for such differences are largely unknown [28]. Prescriptions based on physicians’ beliefs that an infection is bacterial, on patients’ signs and symptoms, or on physicians’ experience without timely use of diagnostic tools are known contributing factors. However, some already existing studies were published in 2018 and may no longer reflect the state-of-the-art knowledge, given the rapid developments in clinical practice and in awareness among the public [13], while others focus specifically on doctors-in-training [14], in-hospital prescribing [15], or the primary care setting [16]. Therefore, an up-to-date review adopting a broader approach—including all medical doctors and all types of care (primary, secondary, and tertiary)—was called for.

## 2. Methods

### 2.1. Study Design

This systematic review protocol was based on the Preferred Reporting Items for Systematic Reviews and Meta-Analyses (PRISMA) [40].

### 2.2. Eligibility Criteria

The inclusion criteria for articles were as follows: (1) the language of publication must be in English or Dutch; (2) the study served as a primary data source; (3) the study was conducted in the Netherlands or in a country elsewhere entirely situated in Europe; (4) the study focused on psycho-socio-organisational reasons for prescribing antibiotics; (5) the study encompassed primary, secondary, and tertiary care settings; (6) the study delved into the physician’s perspective; and (7) the study centred around the prescription of antibiotics. The exclusion criteria included the following: (1) studies exclusively addressing out-of-office prescriptions, as in the Netherlands patients normally go to their own GP, except in exceptional circumstances; (2) studies about the physicians’ perspective of an intervention; and (3) studies of low quality or at high risk of bias. The definition retained for a physician in this study is “one who prescribes antibiotics on a regular basis in first-, second- or third-line care.” All physicians were included regardless of their age, gender, or ethnicity.

### 2.3. Search Strategy

To formulate the search query, appropriate keywords were chosen for each element, resulting in the keywords ‘antibiotics’, ‘potentially inappropriate prescribing’, and ‘Netherlands and Europe’. Then, synonyms for the keywords were formulated in close collaboration with both an information specialist, as well as two domain experts (clinical microbiologists). The final search string was:

antibiotic* OR antimicrobial* OR antibacterial OR antifungal OR “antibacterial” OR “anti-biotic*” OR “anti-microbial*” OR “anti-fungal”ANDrisk-avers* OR “uncertainty avoidan*” OR “inappropriate* withholding” OR prescrib* OR prescrip* OR treat*ANDdeterminant* OR predictor* OR factor* OR influenc*ANDNetherland* OR Dutch OR “the Netherlands” OR Europe

The systematic review was conducted in the electronic databases PubMed and Scopus. The above-mentioned uniform search strategy was used across the different databases. The databases were searched for publications between 1 January 2000 and the date of search (8 March 2023). This was repeated on 30 January 2024 to scan for new research since the original search (focusing on papers published between 8 March 2023 and 30 January 2024).

### 2.4. Study Records

#### 2.4.1. Data Management and Selection Process

Active learning for Systematic Reviews (ASReview version 1.2; open source https://asreview.nl/ (accessed on 21 March 2024)) was used during the initial screening phase of the reviews, for which default settings were used (classifier: Naïve Bayes; Query strategy max; Feature extraction: Term Frequency-Inverse Document Frequency) [41]. ASReview LAB is a software that uses active learning to make reviewing much faster through artificial intelligence (AI) [41]. Before screening, duplicates were removed through the review management programme Covidence [42]. The input for the machine learning algorithm consisted of prior knowledge on five (according to two independent researchers CL and NB) relevant papers and five (according to two independent researchers, CL and NB) irrelevant papers. To ensure that both reviewers trained the same active learning model, both reviewers selected the same articles as prior knowledge.

During the screening phase, ASReview displays potentially relevant articles first and potentially irrelevant articles last. Since the probability of potentially irrelevant articles increases when more articles have been screened, a pre-defined stopping rule can be used to standardise the decision to cease screening. In this review, the data-driven criterion of excluding 100 articles in a row was used [41]. In the first-stage screening, titles and abstracts were screened in ASReview by two independent reviewers (CL and NB) until the stopping rule was met. Remaining articles, which the algorithm deemed irrelevant, were excluded from the review, as is common practice in working with ASReview [41]. Disagreements between the two reviewers were resolved by consultation until mutual agreement was reached. After title and abstract screening, the potentially relevant references were transported back to Covidence, which was used to screen the full texts.

The results of the second search were manually screened on title and abstract in Covidence by one of the authors (NB) and checked by a second (CL), using the same inclusion and exclusion criteria as the original search.

#### 2.4.2. Quality of Evidence

The quality and risk of bias of each publication were evaluated through appropriate checklists for the different study designs by the first author. For observational studies, the Strengthening the Reporting of Observational Studies in Epidemiology (STROBE) checklist was used for quality, and for the risk-of bias, the Newcastle-Ottawa Scale (NOS) was used. The quality and risk of bias of the cross-sectional studies are evaluated by using the Appraisal tool for Cross-Sectional Studies (AXIS) checklist. For qualitative studies, the Consolidated Criteria for Reporting Qualitative Research (COREQ) checklist was used for quality, and the Joanna Briggs Institute (JBI) was used for determining the risk of bias. When certain information was not reported in the included paper, ‘N/A’ was noted in the checklist [43]. Publications that scored ‘low’ on the quality- or ‘high’ risk of bias-checklists were excluded.

#### 2.4.3. Data Collection Process

The standard Covidence data extraction form was adapted for the purpose of this review [44]. The adapted extraction form was pilot tested with four studies, based on which the form was modified to its final version. The extracted data covered: (1) General information (e.g., publication year, country studied); (2) Methods; (3) Participants; (4) Analysis; (5) Results; and (6) Other (study funding sources, conflicts of interest, and ethical approvement). The data extraction form (see Supplement S1) was discussed with and approved by all authors, and the data extraction was performed by CL.

#### 2.4.4. Outcomes

The primary outcomes of the systematic review were psychological, personal, and organisational factors that physicians perceived to be related to their antibiotic prescribing. In addition, cultural differences were examined as secondary outcomes.

### 2.5. Data Synthesis

Because of the prevalently qualitative nature of the literature outcomes, data synthesis is through a narrative summary, allowing for the capture and presentation of the key themes, patterns, and insights [45]. Study findings and themes were extracted from the identified studies during the synthesis process. Analysis of the data revealed similarities, differences, and recurring patterns. The data synthesis was performed by the first author.

## 3. Results

**Figure 1:**
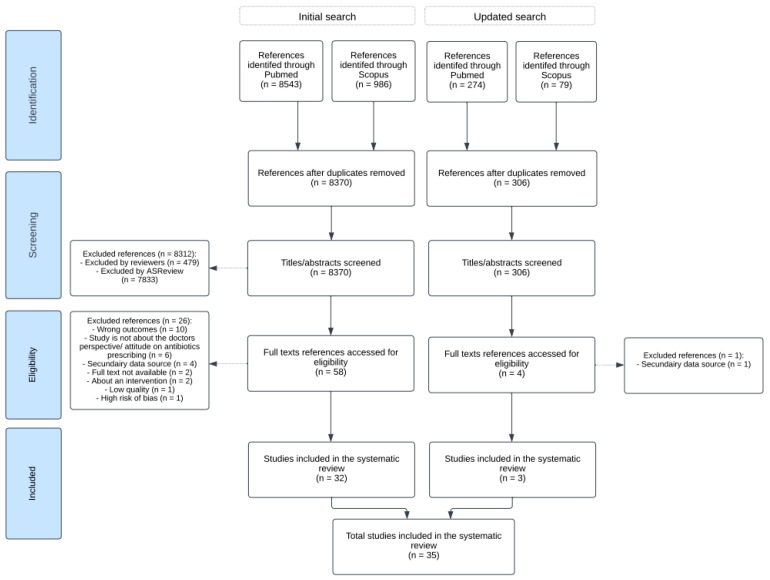
PRISMA flowchart illustrating stages of the literature search and systematic review [40].

The search term resulted in 8543 hits in PubMed and 986 in Scopus. The inter-rater reliability in the first stage of screening between the two independent reviewers was ‘substantial’ (Cohen’s kappa = 0.70) [46]. The second search resulted in 353 hits. After that, four papers were read in full text, and three were found relevant [47,48,49]. See Figure 1 for the PRISMA flowchart.

### 3.1. The Use of ASReview

For the initial search, both reviewers applied the stop rule of 100 articles in a row. As a result, a total of 961 (11%) titles and abstracts were manually screened by the reviewers. The remaining 7409 (89%) titles and abstracts were included or excluded based on ASReview’s algorithm.

### 3.2. Overview of the Included Studies

Of the 58 potentially relevant articles, 26 were excluded due to the exclusion criteria. The updated search resulted in three more articles. Finally, 35 articles, originating from 24 different European countries, were included in the systematic review. It is noteworthy that the studies were conducted mainly in Western Europe, and almost no studies were conducted in Eastern Europe. A visual representation of the geographic distribution of the studied countries is provided in Supplement S2. The study designs of the included papers were qualitative [47,48,49,50,51,52,53,54,55,56,57,58,59,60,61,62,63,64,65,66], observational [67,68] (including cross-sectional [69,70,71,72,73,74,75,76,77,78,79,80]), and cohort [81] studies.

Most [21] papers were related to primary care [47,48,49,51,53,54,55,56,57,59,60,61,64,65,66,68,71,72,74,75,81], four to secondary care [50,52,58,63], one to tertiary care [67]. In addition, four papers combined secondary and tertiary care [70,72,73,79,80], three combined primary care and secondary care [62,77,78], and two related to all care settings [69,76].

The majority of papers [23] covered AB prescribing in general [47,49,50,51,52,54,56,57,59,60,62,63,65,67,69,70,71,73,76,77,80,81]. The other papers focused on a particular class of infections: six focused on respiratory tract infection (RTI) [61,64,66,72,74,78], two on upper respiratory tract infections (URTI) [75,79], one on acute respiratory tract infections (ARTIs) [55], one on urinary tract infections (UTI) [58], one on acute sinusitis [48], and one on acute otitis media (AOM), acute sore throat, rhinosinusitis, and acute cough [68]. See Table 1 for a summary of the characteristics of the included studies. See Supplement S3 for an overview of the quality and the risk of bias in the articles.

### 3.3. Defining Potentially Inappropriate Prescribing

Most studies defined inappropriate prescribing as too many prescriptions or prescribing inconsistent with guidelines but lacked explicit criteria to measure this [56,57,58,59,62,64,65,71,72,76,80]. Only the studies of Lévin et al. [69] and Sikkens et al. [67] have differentiated inappropriate antibiotics in their studies using several criteria. For Lévin et al. [69], these were following local guidelines: switching to oral therapy when possible, prudently prescribing, prescribing the most fitting antibiotic regardless of needing approval, and choosing the antibiotic rationally. For Sikkens et al. [67] a prescription of an antimicrobial was considered appropriate if one of three conditions held true: “(i) it followed relevant guidelines; (ii) it deviated from the relevant guidelines but rational arguments for deviation were documented in the patient file or supplied by the ward physician; and (iii) there was no relevant guideline but the prescription was considered a rational choice”.

### 3.4. Classifying Factors Associated with Potentially Inappropriate Prescribing

Three kinds of factors were associated with PIP: (1) personal factors, (2) psychological factors, and (3) organisational factors (see Table 2 for an overview of all factors found in the literature). Each of these classes of factors will be described and discussed in more detail below. All these classes of factors were found in studies focusing solely on primary [47,48,49,51,53,54,55,56,57,59,60,61,64,65,66,68,71,72,74,75,81] or solely on secondary care [50,52,58], but only personal factors were found in the study focusing solely on tertiary care [67]. However, all classes were acknowledged in studies combining primary with secondary [62,77,78], secondary with tertiary [70,73,79,80], or all three forms of care [69,76]. This means that no factors exclusively hold true for a single form of care. In addition to that, no relevant changes were found in the classes of factors covered in published papers over time. For example, all classes of factors are covered in papers published in the first five years of this review (2000–2005), as well as those published in the last five years of this review (2019–2024).

### 3.5. Personal Factors

Personal factors refer to any characteristics of the prescribing physician themselves. The personal factors found were; work experience, knowledge, and the use of guidelines. Each of these will be elaborated on below.

#### 3.5.1. Work Experience

The work experience of a prescribing physician is often mentioned as being related to antibiotic prescribing [47,48,50,51,52,53,54,55,56,57,58,66,67,69,70,73,74]. Physicians often use their own experience when deciding whether to prescribe antibiotics or not [48,50,51,52,53,69,70]. Beović et al. [70] and Lévin et al. [69] conducted a cross-sectional study with 2366 and 612 young physicians. The studies concluded that 42% and 36.1% used their own experience when deciding whether to prescribe antibiotics or not. Physicians with more experience tend to prescribe fewer antibiotics [54,55,56,57,58,67,73]. Although one study, by Akkerman et al. [74], found the opposite, with experienced GPs prescribing more antibiotics than those less experienced, especially in combination with little knowledge and the feeling of time pressure.

In addition to a direct association with prescribing behaviour, it is noteworthy that Salm et al. [71] concluded that GPs with more than 25 years of work experience assumed that their individual prescribing behaviour has less influence on the development of AMR than their colleagues with less than 7 years of experience (95% CI [0.17–0.62], *p* < 0.001).

#### 3.5.2. Knowledge

Prescribing physicians’ knowledge was also often mentioned as an important factor associated with deciding to prescribe antibiotics [47,50,51,52,53,55,59,60,61,67,69,74,75,76,77,81]. To prescribe antibiotics correctly, prescribers must be aware of the relationship between antibiotic prescribing and resistance, which is often lacking [50,51,59,67,74,75]. Physicians with high levels of education and junior physicians with good perceptions of antibiotic knowledge were more likely to explain appropriate practices related to adverse events by 3.71 times (95% CI [2.09–6.61]) and 1.70 times (95% CI [1.11–2.58]) [69]. In the study of Simpson et al. [59], only a few GPs suggested they needed to update their microbiology knowledge and antibiotic prescriptions. Physicians did see antibiotic resistance as a global problem, but several studies had shown that only around 70% (239/340, 162/214, 2444/3492) saw it as a problem in their workplaces [71,76,77]. Some studies stated that antibiotic prescribing was influenced by a hierarchical system and that the behaviour and/or knowledge of senior physicians were adopted by junior physicians [50,52,53]. In addition to knowledge about correct antibiotic prescribing and resistance, knowledge of existing prescribing guidelines [61] and access to their prescribing statistics [47] may also be important. Finally, the article by Ghigha, et al. [47] confirms that physicians’ knowledge needs to be refreshed regularly.

#### 3.5.3. Use of Guidelines

Using guidelines was also mentioned as a factor that plays a role in physicians’ antibiotic prescribing behaviour [47,48,49,50,51,53,56,61,62,63,66,68,69,71,73], the use of guidelines can help reduce unnecessary antibiotic prescribing [51,62]. Most physicians stated that they follow the available guidelines [69,73]. Although guidelines exist, physicians recognised that prescribing antibiotics is often a subjective process [48,62]. Sometimes physicians made their own ‘guidelines’ instead of using local or national guidelines, or they used the guidelines only to decide which antimicrobial to use [53,62]. A Dutch study showed that physicians did not always agree with the guidelines [63]. In addition, Ghiga et al. [47] and Thaulow [48] also mentioned that the guidelines should be reassessed. Salm et al. [71] stated that the use of guidelines among GPs under 40 years of age was greater than among those over 60 years old (OR 3.97, 95% CI 1.32–11.91; *p* = 0.001). This statement is consistent with the statement of Hampton et al. [62] that contradicting guidelines were more likely to be noticed by senior clinicians than by junior clinicians, which they relate to senior doctors prescribing based on ‘a gut feeling, […] a subjective decision’.

### 3.6. Psychological Factors

In addition to general physician-focused factors, psychological factors were also identified as factors in antibiotic prescribing. The definition of psychological factors that has been retained in this study is “traits and behaviours that derive from people’s personality traits”, in this case, the prescribing physician’s traits and behaviour. The most mentioned psychological factors were (Section 2.1) uncertainty avoidance and (Section 2.2) perceived patient and/or parental pressure. Both will be elaborated upon below.

#### 3.6.1. Physicians ’Attitudes towards (Diagnostic) Uncertainty

Uncertainty avoidance was mentioned as an important psychological factor in many articles [47,48,49,51,53,54,56,57,58,60,62,63,64,66,70,73,78,79]. The articles defined uncertainty avoidance as the fear, anxiety, and overcautiousness about leaving a bacterial infection untreated and/or developing complications [51,58,62]. More experience and better knowledge provided more confidence in deciding whether antibiotics were appropriate or not, but physicians felt most confident when their decision was supported by microbiology laboratory results [53,54,57,58,60,64,73]. More confidence can reduce diagnostic uncertainty [53,60]. Due to the discomfort and uncertainty avoidance, physicians prescribed antibiotics to be on the safe side or prescribed broad spectrum antibiotics to be sure that the infection of the patient was cured [63,77]. Another method used by some physicians to deal with uncertainty was delayed prescriptions [51,55,56,64,66]. In the study conducted by Salm et al. [71] 44% (151/340) of the GPs stated that when it was just before the weekend and it was uncertain how an infection would progress, an antibiotic was prescribed without a strong indication. Conversely, the study by Geitona et al. [80] stated that 74% (204/275) of the paediatricians (in training) never or barely felt uncertainty. Additionally, 65.1% (179/275) of their respondents suggested that the implementation of special guidelines and protocols, as well as the use of diagnostic rapid tests (60%, 165/275), could help reduce diagnostic uncertainty.

#### 3.6.2. Perceived Patient and/or Parental Pressure to Prescribe Antibiotics

How the physician experiences patient pressure is person-dependent [60]. Many of the included studies found that some physicians experienced conflicts when they do not prescribe antibiotics, which they felt impacted the doctor-patient relationship [47,49,53,54,57,58,60,62,64,66,71,72,75]. To maintain a good doctor-patient relationship, physicians sometimes use delayed prescriptions [64]. GPs in Romania [47] and paediatricians in Italy [72,78] stated that part of their experienced patient pressure is caused by the fact that their patients can easily switch to another GP. At the same time, some other studies stated that physicians were not influenced by patient pressure and dissatisfaction [55,72,81]. The slightly disparate results may be explained by the fact that perceived patient and/or parental pressure is often subjective [60,62]. Additionally, parent demand is not always perceived correctly [78]. One study among paediatricians found that in 24% of cases, paediatricians thought parents were expecting antibiotics when parents themselves indicated they were not [78]. The study by Ciofi et al. [72] stated that in 77.1% (611/792) cases, paediatricians said they were not influenced by parents’ expectations. Despite this statement, the same study found that the relative risk of getting antibiotics when parents were seen as “somewhat” expecting it was 2.2 compared to parents who were seen as not expecting it [72]. Finally, better knowledge among patients/parents [51,55] and a good patient-doctor relationship [47] may lead to less demand for antibiotics.

### 3.7. Organisational Factors

The organisational factors identified in the included articles were: (Section 3.1) time and work pressure, and (Section 3.2) diagnostic tests and ease of follow-up. These factors are described below.

#### 3.7.1. Time and Work Pressure

The most mentioned organisational factor that plays a role in prescribing antibiotics was time and work pressure [48,53,54,57,58,60,62,64,65,66,71,81]. According to two articles, physicians working in the emergency room are more likely to prescribe antibiotics incorrectly due to high work pressure [61,81]. The results of Teixeira et al. [81] also show that “with a decrease of one patient per day”, the probability of being a good prescriber increased by 3% (OR [95% CI] = 0.97 [0.94–1.00]; *p* < 0.05). Physicians report that, when they experience work pressure, prescribing antibiotics is easier and faster than explaining why antibiotics are not given [54,57,58,62,64,65,66,71]. In addition, work pressure also negatively impacted their perception of having time for peer consultations [53].

#### 3.7.2. Availability of Diagnostic Tests and Follow-Up

In some of the included articles, prescribing physicians encountered situations where access to diagnostic tests was limited or where it took 3 to 5 days for the results to become available, a duration that could be extended further, particularly during weekends [63,73]. Björkman et al. [65] and Petursson et al. [57] stated that antibiotics were prescribed more often if prescribing physicians were, for some reason, unable to follow up on patients.

## 4. Discussion

**Figure 2:**
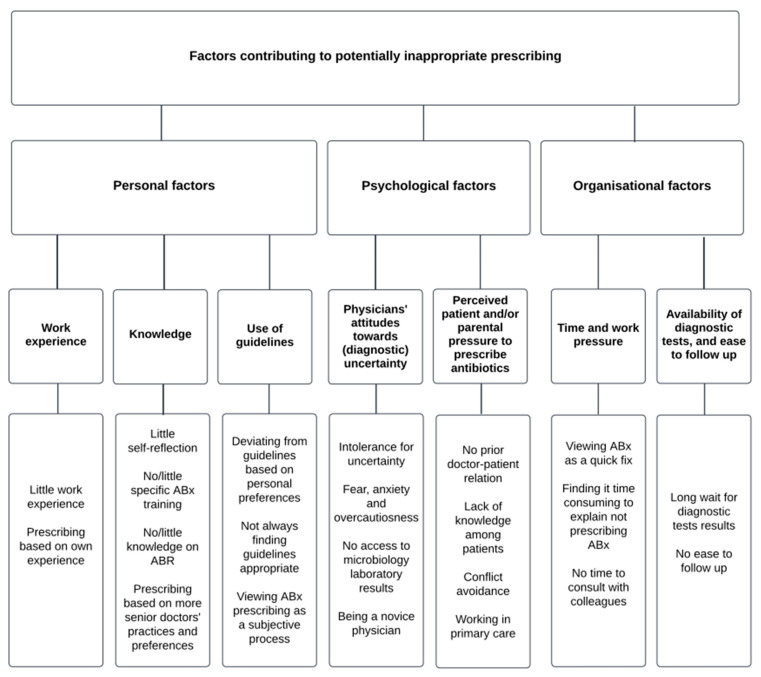
Overview of all the personal, psychological, and organisational factors identified in this study.

This study aimed to investigate the prescribing physician’s perspective on psycho-socio-organisational factors associated with antimicrobial prescribing in primary, secondary, and tertiary care. The identified factors can be divided into personal, psychological, and organisational factors. Personal factors included physicians’ work experience, knowledge, and use of guidelines. Psychological factors concerned physicians’ attitudes towards (diagnostic) uncertainty and perceived patient and/or parental pressure to prescribe antibiotics. Organisational factors encompassed time/work pressure, availability of diagnostic tests, and ease of patient follow-up; see Figure 2 for an overview.

The factors found are largely in line with the systematic review of Sijbom et al. [16]. Physician-related personal factors of work experience and knowledge go hand in hand. Increased work experience relates to enhanced knowledge, contributing to more informed antibiotic prescribing. However, contrary to most studies, the Dutch study by Akkerman et al. [74] regarding RTIs found that physicians with more experience prescribe more antibiotics, especially in combination with little knowledge and the feeling of time pressure. This emphasises that physicians who are more knowledgeable about antibiotics (resistance), regardless of their experience, can make better choices regarding antibiotic prescribing. In addition, they can better explain and substantiate these choices to patients [69]. It is crucial to recognise that improving and broadening knowledge is a pivotal aspect of reducing potentially inappropriate prescribing (PIP). Social norm feedback—which refers to providing physicians with information about the health consequences of antibiotic use—was identified as a potential avenue to raise awareness about the impact of their prescribing behaviour [82]. In addition to knowledge about antibiotics (resistance), knowledge about and adequate use of state-of-the art guidelines is also important [61].

Psychological factors include uncertainty avoidance [51,53,74] and perceived patient/parental pressure. According to the systematic review of Warreman et al. [15] a (lack of) tolerance to uncertainty and fear of adverse outcomes due to untreated infection were prominent determinants of antimicrobial prescribing behaviour. This is in line with the results of the systematic review by Touboul-Lundgren et al. [83], which concluded that there is a correlation between antibiotic use and the cultural dimensions of Power Distance (PD), Uncertainty Avoidance (UA), and Masculinity (MF). On the one hand, having a larger Power Distance may decrease the physicians’ susceptibility to perceived patient pressure. On the other hand, all the studies mentioned above (including our own) acknowledge the relevance of uncertainty avoidance as a relevant factor.

When prescribing physicians encounter uncertainty, they prioritise the immediate treatment of the individual patient over the potential future implications of antibiotic resistance development [62]. This inclination may be attributed to future discounting, defined as “a technique for comparing costs and benefits that occur in different periods. It is independent of inflation and is based on the principle that people prefer to receive goods and services now rather than later” [84]. In the context of antibiotic prescribing, this suggests that physicians are often more inclined to prioritise immediate patient assistance over considering the patient’s potential risk of developing an antibiotic-resistant infection in the future. Additionally, studies [78] have shown that certain organisational factors, such as the availability of rapid testing (e.g., CRP tests), can help reduce inappropriate prescribing due to diagnostic uncertainty and fear of undertreatment [67,72,78]. Next, psychological factors were impacted by the prescribing physicians’ work experience, with novice physicians experiencing more uncertainty [60,76], and their field of work. Finally, with primary care physicians reporting greater patient pressure compared to their counterparts in secondary or tertiary care due to a more active patient role [53,57,60,65,71,73,75,76,78,85]. Similarly, a study showed that healthcare systems may impact the perception of patient pressure as well [67]. For example, the financial systems in Italy make GPs more dependent on their patients’ approval compared to Dutch GPs, which might make Italian GPs more prone to yielding to patient pressure [72,78]. This effect may be bolstered by culturally determined factors. For example, Hofstede suggested that people from different countries/cultures may respond differently to uncertainty. They found that the tolerance of uncertainty in northern European countries is relatively lower compared to southern European countries [86]. So, despite different socio-cultural and organisational backgrounds, uncertainty avoidance plays a role overall, including in the already low-prescribing Netherlands.

Organisational factors encompassed diagnostic testing, ease of patient follow-up, and time/work pressure. In particular, the time/work pressure were found to negatively impact antibiotic prescribing in a multitude of ways. First of all, it was noted to influence decisions because of the ease of prescribing antibiotics, as opposed to explaining why they were not prescribed [53,54,57,58,62,64]. Second, it also has a negative impact on antibiotic prescribing since physicians do not always have time to consult with colleagues [53]. The extent to which prescribing physicians experience time and work pressure may be influenced by organisational factors such as the way shifts are set up (impacting the number of patients under the physicians’ supervision and how easy it is to consult a colleague [67]) and the number of patients a physician is allowed to see in a day [81]. Finally, physicians are less likely to (inappropriately) prescribe antibiotics if they have easy access to fast diagnostic testing in their healthcare system and if they can follow-up on a patient’s health (which can be hindered by a weekend) [65,71].

### 4.1. Tackling Potentially Inappropriate Prescribing

Interventions to improve antibiotic prescribing should be tailored to the different needs and preferences of different (types of) prescribing physicians. Uncertainty avoidance emerged as a key factor underlying many of the factors that play a role in antibiotic prescribing, emphasising the need to address this factor in interventions. Making decisions in uncertain circumstances is an integral part of practising medicine. Yet, to date, existing interventions to improve antibiotic prescribing mostly attempt to reduce uncertainty rather than prepare physicians to deal with or tolerate uncertainty to minimise discomfort [56,87]. Furthermore, interventions targeting physicians were found to be more effective than interventions targeting institutions, highlighting the importance of a personalised approach [82]. Yet, such interventions cannot be assumed to be a standalone, easy fix. They should be accompanied by cultural changes (recognising and embracing tolerance for uncertainty [87]) and organisational measures (recognising and accounting for the complexity and time-consuming characteristics of infectious disease care) [56,57,58,59,60,61,62,63,64,65].

### 4.2. Reflection on the Updated Search

One truly new insight that can be added to the body of literature that was used in the current paper based on the update is that having access to prescribing statistics may be helpful for physicians as a motivation to change their prescribing behaviour [47]. This is in line with prior research into experts’ and physicians’ needs with regard to audit and feedback systems for antimicrobial resistance prevention measures, which subscribed to this apparent need for more insight into their own behaviour [88].

### 4.3. Strengths and Limitations

This study has several strengths that contribute to its value in understanding the psycho-socio-organisational factors influencing potentially inappropriate antimicrobial prescribing among physicians. Firstly, it employs a systematic review method, adhering to PRISMA guidelines, ensuring a robust and transparent selection process. In that sense, the utilisation of AI in the selection process contributes to its accuracy, efficiency, and reproducibility [89]. Focusing specifically on European studies allows the research to be contextualised geographically and culturally. The exclusion of low-quality studies further increased the study’s value. The narrative synthesis method allows for a nuanced understanding of the identified determinants, including work experience, knowledge, guideline adherence, and uncertainty management. Highlighting uncertainty management as a key factor, the study provides actionable insights for educational interventions aimed at reducing potentially inappropriate prescribing.

A possible limitation of ASReview is that ASReview typically finds 95% of the relevant articles, so some relevant articles may have been missed. However, this is a highly acceptable rate considering the average 90% that is achieved through a manual search [90]. In addition, the inclusion of only English or Dutch articles and the use of two databases might introduce some bias. However, in general, language bias has been shown to have minimal impact on the outcomes of systematic reviews [90], and the chosen databases covered a comprehensive range of relevant articles. The majority of these included papers (21/35) based their findings on relatively small sample sizes of less than 50 participants. Given the qualitative nature of many of these studies, this may be highly suitable and does not mean the studies have little value [91]. Yet, it should be kept in mind to ensure that the findings of this review are interpreted with some caution. Furthermore, efforts were made to minimise bias through stringent eligibility criteria [92], risk-of-bias tools, and a two-stage independent review process. Nevertheless, a narrative summary inherently carries subjective interpretations, introducing potential bias [93]. Future research should address these limitations and explore the impact of tailored and personalised interventions on antibiotic prescribing practices.

## 5. Conclusions

A comprehensive exploration of factors associated with antibiotic prescribing has revealed the importance of personal, psychological, and organisational factors. Differences, including cultural differences, within health care systems play a role in these factors. To improve antibiotic prescribing, support from patients, organisations, and the healthcare system are required, underscoring the complexity of the challenges faced by individual physicians.

We found a substantial body of evidence to corroborate the notion that uncertainty avoidance is one of the most important factors influencing antibiotic prescribing. Next to that, work experience, knowledge, and the use of guidelines were important factors influencing antibiotic prescribing. On the one hand, measures should be taken to reduce this uncertainty. Prescribing physicians experience the least uncertainty when their decisions are supported by microbiology laboratory results, yet the impracticality of certain tests for patients and the time pressure to get swift results highlight the need for alternative solutions. The point-of-care CRP test, in this regard, provides a viable solution as it is a rapid yet sensitive test that can be used to confirm physicians’ suspicion of an infection, thus reducing uncertainty while keeping the added time pressure to a minimum. On the other hand, measures should be taken to improve physicians’ ability to optimally cope with uncertainty. Work experience and knowledge are important factors that helped to mitigate uncertainty, emphasising the importance of continuous education for physicians throughout their careers. In addition, fostering physicians’ self-reflection and improving their skills to manage uncertainty are vital components of this approach. Reduced uncertainty could reduce inappropriate prescribing due to perceived patient and/or parental pressure. Lastly, from a health care system or organisational perspective, it is important to allocate sufficient time per patient to reduce time and work pressure, thereby aiding in reducing potentially inappropriate prescribing (PIP).

In light of these findings, a multifaceted strategy that integrates education, self-reflection, and systemic support—e.g., minimising experienced time pressure and optimising the availability of fast diagnostic testing—is essential to improve antimicrobial prescribing practices and mitigate the impact of various contributing factors.

## Figures and Tables

**Table 1:** Summary of characteristics of the included studies.

Author	Country	Publication Year	Study Design	Setting	Infection	Physicians (Male/Female)	Mean Age Physicians	Mean Years of Experience of Physicians
Akkerman AE et al. [74]	NL	2005	Cross-sectional	Primary care	RTI	84 (57/27)	N/A	16
Beovíc B et al. [70]	Sweden, UK, Spain, Greece, France, Italy, Croatia, Portugal, Slovenia, Austria, Bulgaria, and Turkey	2019	Cross-sectional	Secondary and tertiary care	Any	2366 (883/1483)	N/A	N/A
Björkman I et al. [65]	Iceland	2002	Qualitative	Primary care	Any	20 (13/7)	N/A	N/A
Björndóttir I et al. [53]	Iceland	2002	Qualitative	Primary care	Any	10 (8/2)	48	N/A
Brookes-Howell L et al. [60]	UK, Spain, Hungary, Poland, Norway, Italy, and Belgium	2012	Qualitative	Primary care	Any	80 (47/33)	43	16
Carlsson F et al. [49]	Sweden	2022	Qualitative	Primary care	Any	267 (N/A)	48	N/A
Charani E et al. [52]	UK	2013	Qualitative	Secondary care	Any	10 (N/A)	N/A	N/A
Ciofi degli Atti ML et al. [72]	Italy	2006	Cross-sectional	Primary care	RTI	2151 (N/A)	N/A	N/A
De Souza V et al. [50]	Ireland	2006	Qualitative	Secondary care	Any	18 (8/10)	34.5	N/A
Dekker AR et al. [68]	NL	2015	Observational	Primary care	AOM, acute sore throat, rhinosinusitis, and acute cough	48 (N/A)	N/A	N/A
Eyer MM et al. [58]	CH	2016	Qualitative	Secondary care	UTI	21 (N/A)	N/A	N/A
Geitona M et al. [80]	Greece	2015	Cross-sectional	Secondary care and tertiary care	Any	275 (97/178)	37.2	N/A
Ghiga I et al. [47]	Romania	2023	Qualitative	Primary care	Any	12 (7/5)	N/A	N/A
Grossman Z et al. [79]	Germany, Spain, and Italy	2012	Cross-sectional	Secondary care and tertiary care	URTI	685 (359/326)	50.9	N/A
Hampton T et al. [62]	UK	2021	Qualitative	Primary care and secondary care	Any	21 (N/A)	N/A	N/A
Horwood J et al. [64]	UK	2016	Qualitative	Primary care	RTI	22 (5/17)	N/A	N/A
Lévin C et al. [69]	France	2018	Cross-sectional	Primary care, secondary care, and tertiary care	Any	641 (281/360)	N/A	N/A
Moro ML et al. [78]	Italy	2009	Cross-sectional	Primary care and secondary care	RTI	633 (244/389)	48	17
Petursson P et al. [57]	Iceland	2005	Qualitative	Primary care	Any	16 (13/3)	N/A	N/A
Poss-Doering R et al. [56]	Germany	2020	Qualitative	Primary care	Any	27 (18/9)	N/A	26
Rousounidis A et al. [75]	Cyprus	2011	Cross-sectional	Primary care	URTI	33 (22/11)	N/A	N/A
Ryves R et al. [66]	UK	2016	Qualitative	Primary care	Any	32 (N/A)	N/A	N/A
Saliba- Gustafsson EA et al. [55]	Malta	2021	Qualitative	Primary care	ARTIs	20 (14/6)	52	26
Salm F et al. [71]	Germany	2018	Cross-sectional	Primary care	Any	340 (128/212)	51.9	16.7
Schouten JA et al. [63]	NL	2006	Qualitative	Secondary care	Any	18 (8/10)	34.5	N/A
Sikkens JJ et al. [67]	NL	2018	Observational	Tertiary care	Any	150 (N/A)	N/A	N/A
Simões AS et al. [73]	Portugal	2018	Cross-sectional	Secondary care and tertiary care	Any	30 (13/17)	30	N/A
Simpson SA et al. [59]	UK	2006	Qualitative	Primary care	Any	40 (29/11)	N/A	N/A
Spernovasillis N et al. [76]	Greece	2019	Cross-sectional	Primary care, secondary care, and tertiary care	Any	214 (86/128)	30.1	4.6
Strandberg EL et al. [61]	Sweden	2013	Qualitative	Primary care	RTI	13 (3/10)	N/A	N/A
Thaulow J et al. [48]	Norway	2023	Qualitative	Primary care	Acute sinusitis	25 (5/20)	42	11.3
Teixeira Rodrigues A et al. [81]	Portugal	2016	Cohort longitudinal	Primary care	Any	421 (207/214)	55	N/A
Velasco E et al. [77]	Germany	2011	Cross-sectional	Primary care and secondary care	Any	3492 (2222/1200)	N/A	N/A
Van der Zande MM et al. [54]	UK	2019	Qualitative	Primary care	Any	41 (18/23)	N/A	N/A
Vazquez-Lago JM et al. [51]	Spain	2011	Qualitative	Primary care	Any	33 (19/14)	N/A	N/A

Footnote: respiratory tract infection (RTI), upper respiratory tract infection (URTI), acute respiratory tract infections (ARTIs), urinary tract infections (UTI), and acute otitis media (AOM). N/A denotes that this kind of information was not reported in the publication. UK: United Kingdom. CH: Switzerland. NL: The Netherlands.

**Table 2:** Overview of the factors found in the literature.

Author	Work- Experience	Knowledge	Use of Guidelines	Uncertainty Avoidance	Perceived Patient and/or Parental Pressure	Time and Work Pressure	Diagnostic Tests and Follow-up
Akkerman AE et al. [74]	●	●					
Beovic B et al. [70]	●			●			
Björkman I et al. [65]					●	●	●
Björndottir I et al. [53]	●	●	●	●	●	●	
Brookes-Howell L et al. [60]		●				●	
Carlsson F et al. [49]			●	●	●		
Charani E et al. [52]	●	●					
Ciofi defli Atti ML et al. [72]					-		
De Souza V et al. [50]	●	●	●				
Dekker AR et al. [68]			●				
Eyer MM et al. [58]	●			●		●	
Geitona M et al. [80]				-			
Ghiga I et al. [47]	●	●	●	●	●		
Grossman Z et al. [79]				●			
Hampton T et al. [62]			●	●		●	
Horwood J et al. [64]				●		●	
Lévin C et al. [69]	●	●	●				
Moro ML et al. [78]				●	●		
Petursson P et al. [57]	●			●	●	●	●
Poss-doering R et al. [56]	●		●	●			
Rousounidis A et al. [75]		●			-		
Ryves R et al. [66]	●		●	●	●	●	
Saliba Gustafsson EA et al. [55]	●	●			-		
Salm F et al. [71]			●		●	●	
Schouten JA et al. [63]			●	●			●
Sikkens JJ et al. [67]	●	●					
Simões AS et al. [73]	●		●		●		●
Simpson SA et al. [59]		●					
Spernovasilis N et al. [76]		●			●		
Strandberg EL et al. [61]		●	●				
Teixeira Rodrigues A et al. [81]		●			-	●	
Thaulow J et al. [48]	●		●	●	●	●	
Velasco E et al. [77]		●		●			
Van der Zande MM et al. [54]	●			●	●	●	
Vazquez-Lago JM et al. [51]	●	●	●	●	●		

● Indicates that the paper found and supports the factor. - Indicates that the paper did address the factor, but in a contradictory direction from others (e.g., explicitly stating it had no impact). Empty cells indicate that the paper did not find or address the factor.

## Data Availability

Dataset available on request from the authors.

## Supplementary Materials

The following supporting information can be downloaded at: https://www.mdpi.com/article/10.3390/idr16040051/s1, S1: Data extraction form; S2: Overview of geographic distribution of included papers; S3: Overview of Risk of Bias of the included articles.
