# Supplementary material for: Why Do Physicians Prescribe Antibiotics? A Systematic Review of the Psycho-Socio-Organisational Factors Related to Potentially Inappropriate Prescribing of Antimicrobials in Europe
Source: Infect Dis Rep. 2024 Jul 25;16(4):664–83. doi: 10.3390/idr16040051 (PMC11353809; doi:10.3390/idr16040051)
Supplement: Supplementary file 1 [file idr-16-00051-s001.zip › idr-3069525-supplementary.pdf]

Supplement S1

Data items

General information

Study ID

Title

Abstract

Publishing date

County in which the study is conducted;

- Sweden
- UK
- Netherlands
- Germany
- Spain
- Malta
- Greece
- Belgium
- France
- Italy
- Other

Notes

Methods

Aim of study

Study design;

- Cohort study
- Observational study
- Cross-sectional study
- Qualitative study
- Other

Start date and end date

Participants

The total number of participants invited

The total number of participants who participate

How many participants were excluded and why?

The setting of the study;

- Primary care
- Secondary care
- Tertiary care
- Other

Population description

Inclusion criteria

Exclusion criteria

Method of recruitment of participants;

- Phone
- Mail
- Clinic patients
- Voluntary
- Unknown
- Other

Method to conduct the interview/questionnaire;

- Face-to-face

- Phone
- Mail
- Online call
- Written questionnaires
- Online questionnaires
- Unknown
- Other

Analysis

Which method is used for analysing?

Results

Main outcomes

Which non-medical determinants influence antibiotic prescribing?

	Determinant	Target population	For which disease	Relating to	Significant effect	Mean	Confidence interval	Percentage
1								
2								
3								
4								
5								

Discussion outcomes

Other

Study funding sources

Conflicts of interest

Ethical approval

Supplement S2

The GEOGRAPHIC distribution of the frequency that a country is included in the articles used.

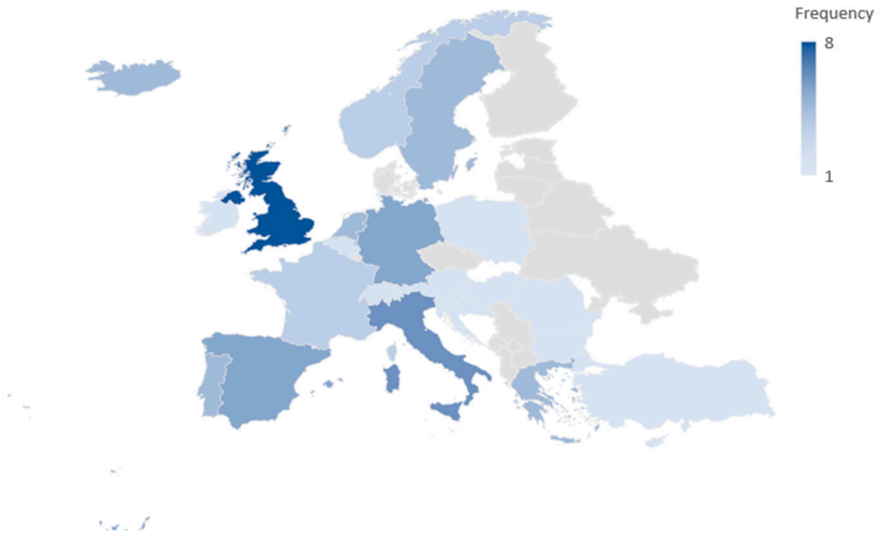

*Footnote: *n exceeds total number of papers, as some were executed in multiple countries.*

United Kingdom	8
Italy	5
Germany and Spain	4
Iceland, Portugal, Sweden, Netherlands, and Greece	3
Norway and France	2
Austria, Bulgaria, Turkey, Romania, Slovenia, Croatia, Switzerland, Republic of Ireland, Malta, Cyprus, Belgium, Poland, and Hungary	1

Supplement S3

An overview of the quality and the risk of bias in the articles

Author	Quality	Risk-of-bias	Reference number
Ghiga I, et al.	19/32 moderate	10/10 low	47
Thaulow J, et al.	20/32 moderate	10/10 low	48
Carlsson F, et al.	20/32 moderate	9/10 low	49
De Souza V, et al.	18/32 moderate	8/10 low	50
Vazquez-Lago JM, et al.	19/32 moderate	9/10 low	51
Charani E, et al.	24/32 high	9/10 low	52
Björndóttir I, et al.	18/32 moderate	7/10 moderate	53
Van der Zande MM, et al.	21/32 moderate	10/10 low	54
Saliba- Gustafsson EA, et al.	17/32 moderate	10/10 low	55
Poss-Doering R, et al.	19/32 moderate	8/10 low	56
Petursson P, et al.	19/32 moderate	7/10 moderate	57
Eyer MM, et al.	24/32 high	9/10 low	58
Simpson SA, et al.	21/32 moderate	10/10 low	59
Brookes-Howell L, et al.	24/32 high	9/10 low	60
Strandberg EL, et al.	15/32 moderate	8/10 low	61
Hampton T, et al.	27/32 high	10/10 low	62
Schouten, et al.	20/32 moderate	9/10 low	63
Horwood J, et al.	25/32 moderate	9/10 low	64
Björkman I, et al.	22/32 high	8/10 low	65
Ryves R, et al.	19/32 moderate	9/10 low	66
Lévin C, et al.	16/20 high	16/20 low	67
Beovíc B, et al.	15/20 moderate	15/20 low	68
Salm F, et al.	15/20 moderate	15/20 moderate	69
Ciofi degli Atti ML, et al.	13/20 moderate	13/20 moderate	70
Simões AS, et al.	14/20 moderate	14/20 moderate	71

Akkerman AE, et al.	16/20<br>high	16/20<br>low	72
Rousounidis A, et al.	17/20<br>high	17/20<br>low	73
Spernovasillis N, et al.	15/20<br>high	15/20<br>low	74
Valesco E, et al.	18/20<br>high	18/20<br>low	75
Moro ML, et al.	15/20<br>moderate	15/20<br>moderate	76
Grossman Z, et al.	16/20<br>high	16/20<br>low	77
Geitona M, et al.	16/20<br>high	16/20<br>low	78
Sikkens JJ, et al.	28/34<br>high	7/9<br>low	79
Dekker AR, et al.	22/34<br>high	8/9<br>low	80
Teixeira Rodrigues A, et al.	28/32<br>high	8/9<br>low	81
